# Interfacial Transition Zone Strengthening in Aeolian Sand Concrete via ssDNA Anchored CNTs on Alkali-Activated Surface Layer

**DOI:** 10.3390/ma19051023

**Published:** 2026-03-06

**Authors:** Yi Zhou, Taotao Cai, Xingu Zhong, Chao Zhao, Tianye Luo, Kunlong Tian, Yuanyuan Li

**Affiliations:** Hunan Provincial Key Laboratory of Structures for Wind Resistance and Vibration Control & School of Civil Engineering, Hunan University of Science and Technology, Taoyuan Road, Yuhu District, Xiangtan 411201, China

**Keywords:** aeolian sand, interfacial transition zone, ssDNA, alkali activation, CNTs

## Abstract

The use of aeolian sand as a fine aggregate in concrete production provides a sustainable pathway to valorize abundant aeolian resources while alleviating the global shortage of natural construction aggregates. However, the high ultrafine particle content of aeolian sand results in the formation of highly porous interfacial transition zones (ITZ) between sand particles and cement paste, which is the primary cause of the inherent brittleness and inferior mechanical performance of aeolian sand concrete. To overcome this critical limitation, an alkali-activated surface layer (ASL) was constructed on aeolian sand via 4 mol/L KOH activation. This process induced the surface micro-dissolution of minerals to create high-density active ion sites (specifically Ca^2+^, K^+^, Na^+^, and Fe^3+^). These sites facilitated the precise anchoring of carbon nanotubes (CNTs) through the chemical coordination of single-stranded deoxyribonucleic acid (ssDNA). The influence of the ASL and the ssDNA/CNTs nanocomposite on the ITZ was elucidated through macro-mechanical testing and multi-scale microstructural characterization. Experimental results demonstrated that compressive strength, flexural strength, and compressive energy dissipation increased by 48%, 67%, and 42%, respectively. Microstructurally, the modification promoted a pore refinement mechanism, reducing the proportion of harmful (pores > 0.1 μm) from 51% to 20% and narrowing the ITZ width from 20–40 μm to 10–15 μm (a 67% reduction). The observed performance enhancement is attributed to the synergistic effect of the ASL and ssDNA/CNTs, which transforms the inherently weak ITZ into a chemically reinforced interfacial phase via molecular-scale coordination bonding and optimized stacking of cement hydration products.

## 1. Introduction

Concrete, a cornerstone of modern infrastructure, has an annual global consumption of billions of tons, creating immense demand for traditional aggregates such as river sand and crushed stone [1,2,3]. However, the excessive exploitation of these resources has led to severe ecological and environmental problems, including resource depletion and biodiversity loss [4]. Therefore, developing sustainable alternative aggregates to mitigate this environmental pressure has become a critical challenge in the global civil engineering field.

Against this backdrop, aeolian sand, with its abundant in reserves, has emerged as a highly promising alternative fine aggregate [5,6,7]. Although aeolian sand concrete demonstrates excellent performance in terms of strength and engineering applicability [8,9], its widespread application is hindered by a core technical bottleneck: the high ultrafine particle content of aeolian sand results in a porous and low-quality ITZ between the sand and cement paste [5,10]. As an inherent weak link in concrete, the properties of the ITZ directly determine the overall mechanical performance and toughness of the material. Consequently, aeolian sand concrete typically suffers from high brittleness and inferior mechanical properties, which severely restricts its practical use in engineering projects.

To overcome this bottleneck, researchers have attempted to utilize polymers [11,12] and micro-nano materials (such as silica fume and CNTs) [13,14,15,16] to enhance the ITZ. However, existing methods have significant limitations: while polymers can improve toughness, this often comes at the expense of compressive strength and can retard cement hydration [12]. Micro-nano materials, especially CNTs, can enhance both strength and toughness, but the challenge of achieving cost-effective, efficient dispersion and precise positioning within the ITZ remains substantial. Conventional dispersion methods (such as ultrasonic treatment) are prone to causing re-agglomeration and result in a random distribution within the cement matrix [17]. Meanwhile, strategies to achieve precise distribution in the ITZ, such as polymer coating [18,19] or silane modification [20], not only involve complex processes and high costs but may also hinder the direct interaction between CNTs and cement hydration products (calcium silicate hydrate, C-S-H), or lead to excessive strength loss that limits practical application. In summary, there is currently a lack of an effective, low-cost, and simple-process strategy that can achieve uniform dispersion and precise anchoring of reinforcing materials within the ITZ to simultaneously improve the strength and toughness of concrete. This constitutes a critical knowledge gap that current research urgently needs to fill.

Zheng et al. [21] first reported in 2003 that ssDNA can separate carbon nanotube bundles into individual nanotubes with ultrasonic assistance. ssDNA consists of three parts: nitrogenous bases, deoxyribose, and a phosphate backbone. The π-bonds on its nitrogenous bases can combine with the π-bonds on the surface of CNTs, achieving efficient separation of CNTs. Meanwhile, the ssDNA chain also contains abundant amino, carbonyl, and phosphate groups, which can form chemical bonds with various metal ions [22]. This property has been applied in the fields of metal material corrosion protection [23], nanoelectronics [24], and sensors [25]. As a silico-aluminous material, aeolian sand can dissociate its silica and alumina to generate silicates and aluminates in a strong alkaline solution [26]. These silico-aluminates contain a wealth of metal ions, providing potential possibilities for the reaction between ssDNA and aeolian sand [27]. The metal ions released from the alkali-activated surface can serve as active sites, enabling strong chemical coordination with the functional groups of ssDNA [28]. This ensures that the ssDNA/CNTs nanocomposite is firmly anchored on the sand surface via cation-mediated molecular bridging [29,30,31]. In addition, previous research has shown that ssDNA-modified CNTs can significantly enhance the strength of foam concrete, indicating that ssDNA has a positive effect on the performance of cement-based materials [32], which provides an important reference for this study.

To fill this gap, an innovative modification strategy is proposed herein. A KOH solution is utilized to induce surface micro-dissolution of aeolian sand, disrupting stable Si-O-Si bonds to form an ASL rich in active ion sites (Ca^2+^, K^+^, Na^+^, and Fe^3+^) [28,33]. Subsequently, the unique molecular structure of ssDNA is leveraged to achieve precise anchoring of CNTs. This biomolecule facilitates the efficient dispersion of CNTs via π-π stacking while simultaneously forming chemical coordination bonds with the metal ions in the ASL through its functional groups.

The core contribution of this strategy are twofold: first, it proposes a novel in situ ITZ reinforcement method that requires no expensive coatings and has a simple preparation process; second, it creatively solves the long-standing challenges of CNT dispersion and positioning in cementitious materials using the biomolecule ssDNA. Through systematic macro-mechanical testing and multi-scale microstructural characterization, this research aims to elucidate the underlying mechanism for the synchronous enhancement of strength and toughness, thereby providing new theoretical foundations and technical pathways for the large-scale resource utilization of aeolian sand and the development of green, high-performance concrete.

## 2. Materials and Methods

### 2.1. Materials and Sample Preparation

#### 2.1.1. Materials

**Figure 1:**
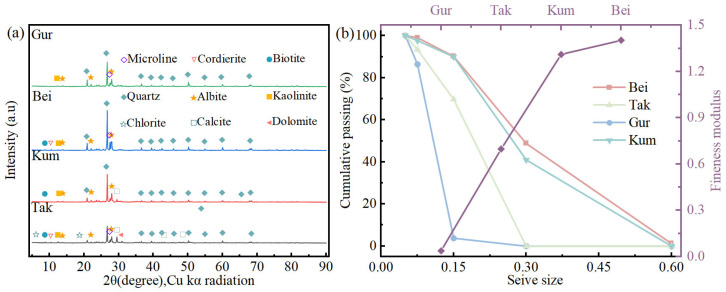
Basic information of the raw materials: (**a**) XRD patterns of aeolian sands from four regions; (**b**) particle size distribution and fineness modulus of the four aeolian sands.

Aeolian sand samples were collected from four deserts in China: Beitun (Bei), Taklamakan (Tak), Gurbantunggut (Gur), and Kumtag (Kum). The X-ray diffraction (XRD) patterns of the four sand samples are presented in Figure 1a, and their main chemical compositions are listed in Table 1. All samples have similar chemical compositions, with silicon dioxide as the primary component. Type P·O 52.5 ordinary Portland cement (OPC), conforming to the Chinese National Standard GB175-2007 [34], was used as the cementitious material for all concrete mixes. KOH with a purity of ≥90% was purchased from Hunan Qilu New Materials Technology Co., Ltd. (Changsha, China). Salmon sperm ssDNA, with a length range of 30–200 deoxyribonucleotides, was used as the dispersant for CNTs, purchased from Beijing Huamaike Biotechnology Co., Ltd. (Beijing, China). Multi-walled carbon nanotubes, with a diameter of 3–15 nm and a length of 15–30 μm, were purchased from Jiangsu Xianfeng Nanomaterials Technology Co., Ltd. (Nanjing, China). A powdered polycarboxylate-based superplasticizer was used to adjust the workability of fresh concrete. The fineness modulus and particle size distribution of the aeolian sand were determined through sieve analysis using square hole sieves. The calculation formula for the fineness modulus is given by Equation (1).

(1) $$M_{x}=\frac{(A_{2}+A_{3}+A_{4}+A_{5}+A_{6})-5A_{1}}{100-A_{1}}$$

where $M_{x}$ is the fineness modulus, and $A_{1},\;A_{2},\;A_{3},\;A_{4},\;A_{5},\;A_{6}$ is the cumulative sieve residue percentage of 4.75 mm, 2.36 mm, 1.18 mm, 0.60 mm, 0.30 mm, 0.15 mm sieve, respectively. The particle size distribution of the four aeolian sands is shown in Figure 1b. The Kum and Bei sands have similar particle sizes, with fineness moduli of 1.31 and 1.402, respectively. The Gur aeolian sand is the finest, with a cumulative sieve residue of 96.2% for particles smaller than 0.15 mm.

#### 2.1.2. Mix Proportion and Sample Preparation of Concrete

The purpose of this study is to explore the effect of ssDNA/CNTs anchoring on the surface of aeolian sand on the ITZ of concrete and its applicability across different regions. All concrete mixes were prepared using aeolian sand as the full replacement for fine aggregate, with a fixed water-cement ratio of 0.22 and a polycarboxylate superplasticizer dosage of 2% by mass of cement. To isolate the specific contributions of the ASL to the mechanical performance of the concrete, the Bei-AA group was established as a control, consisting of aeolian sand treated exclusively with alkali activation in the absence of ssDNA/CNTs. The mix proportions of all concrete groups are detailed in Table 2.

Aeolian sand concrete specimens were fabricated using a JJ-5 cement mortar mixer in accordance with the GB/T 50081-2019 [35]. The mixing procedure was as follows: OPC and aeolian sand were dry-mixed at low speed for 120 s; a superplasticizer solution (prepared with half of the mixing water) was then added, and mixing continued for 60 s; finally, the remaining water was added, and the mixture was mixed for an additional 180 s. After 24 h of natural curing, the specimens were demolded and subjected to a strictly controlled steam curing cycle: an initial 1 h holding period at 20 °C, followed by heating to 90 °C at a rate of 15 °C/h, a 3 d isothermal hold at 90 °C, and final cooling back to 20 °C at the same rate of 15 °C/h.

### 2.2. Anchoring of ssDNA/CNTs on Aeolian Sand Surface

The CNT suspension was prepared through ultrasonic dispersion for 40 min with the assistance of ssDNA, utilizing an ultrasonic instrument (LS-1200/20K, Wuxi Yuanshengte Intelligent Technology Co., Ltd., Wuxi, China). The ultrasonic parameters were set as follows: power of 800 W, frequency of 20 kHz, with an intermittent mode of 10 s of ultrasonication followed by a 3 s pause. To maintain a low temperature during the ultrasonication process, an ice-bath environment was employed, and the ice was replaced every 10 min. A stainless-steel cup was used as the container to facilitate rapid heat transfer to the ice water, ensuring that the solution temperature remained consistently below 8 °C throughout the process, in reference to the study by Zheng et al. [21].

The ssDNA molecules wrapped helically around the surface of CNTs, with the π-bonds of the bases in ssDNA binding to the π-bonds on the CNTs surface. The phosphate backbone of ssDNA dissociated hydrogen atoms in the solution, acquiring a negative charge that prevented CNTs aggregation through charge repulsion. For the aeolian sand, organic impurities such as weeds and branches were removed, and the sand was directly immersed in a 4 mol/L KOH solution (with KOH accounting for 5% of the sand mass [36]) for 1 h. After the initial KOH activation, the aeolian sand was subjected to a thorough triple-washing process with deionized water. This step was essential to remove any excess unreacted alkali and soluble silicates that might interfere with the subsequent hydration process or lead to unwanted salt crystallization. Following the washing, the sand was dried in a 90 °C oven to constant weight. Under the action of KOH, surface oxides such as SiO_2_ and Al_2_O_3_ on the aeolian sand dissolved, generating soluble [SiO_4_]^4−^ and [Al(OH)_4_]^−^ oligomers. Simultaneously, cations including Mg^2+^, Ca^2+^, Fe^3+^, Na^+^, and K^+^ were released. These oligomers and cations diffused into the solution, providing a material basis for subsequent polycondensation reactions. Under the action of 4 mol/L KOH, the surface of aeolian sand underwent micro-dissolution [28], releasing SiO_2_ and Al_2_O_3_ oligomers along with active cations that enriched on the sand surface to form an ASL. This layer providing high-density active sites for subsequent chemical chelation with ssDNA, rather than forming a bulk geopolymer network.

**Figure 2:**
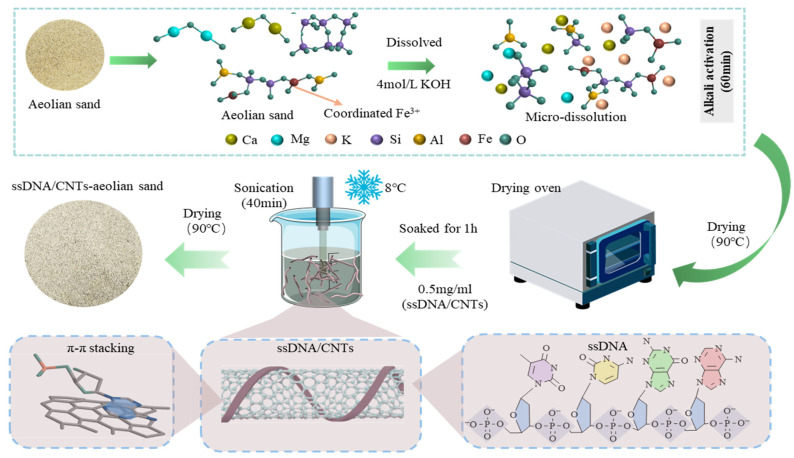
Preparation process of bio-inorganic composite interfacial layer.

After cooling to room temperature, the alkali-activated sand was immersed in the CNT suspension for 1 h. Following the removal of excess CNT suspension, the sand was dried again in a 90 °C oven. Finally, the aeolian sand with anchored CNTs was collected and sealed with plastic wrap to prevent moisture absorption from the air. The entire process is illustrated in Figure 2.

### 2.3. Test Method

To systematically evaluate the synergistic effects of the ASL and ssDNA/CNTs on the performance of aeolian concrete, a multi-scale testing program was executed. This program encompassed macro-mechanical testing and various microstructural characterization techniques to reveal the influence mechanism of the modified sand on the ITZ. A comprehensive summary of the nine experimental methodologies, including specimen specifications, preparation procedures, and key analytical parameters for the equipment used, is presented in Table 3. Except for the Bei-AA specimen, all other specimens completed the micro-test, with a total of 8 specimens participating in the micro-test, and only the mechanical properties of the Bei-AA specimen were tested.

#### 2.3.1. Mechanical Property Testing

**Figure 3:**
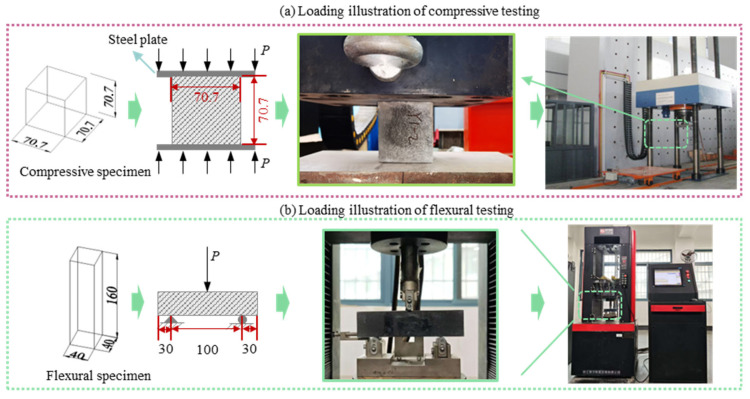
Schematic diagram of mechanical performance test.

In accordance with GB/T 50081-2019 [35], the compressive strength of aeolian sand concrete was tested using 70.7 mm × 70.7 mm × 70.7 mm cubic specimens at a loading rate of 0.5 MPa/s. Flexural strength tests were conducted using 40 mm × 40 mm × 160 mm prismatic specimens at a loading rate of 0.05 MPa/s. A schematic diagram of the test setup is shown in Figure 3. All tests were performed immediately after the specimens cooled to room temperature following curing. A total of 9 groups of mechanical property tests were conducted, with 3 specimens per group. Among them, there were 27 specimens for compressive strength tests and 27 specimens for flexural strength tests, resulting in a total of 54 specimens. Three samples were prepared for each type of specimen, and the average value of the final three samples was used as the test result.

#### 2.3.2. X-Ray Diffraction (XRD)

**Figure 4:**
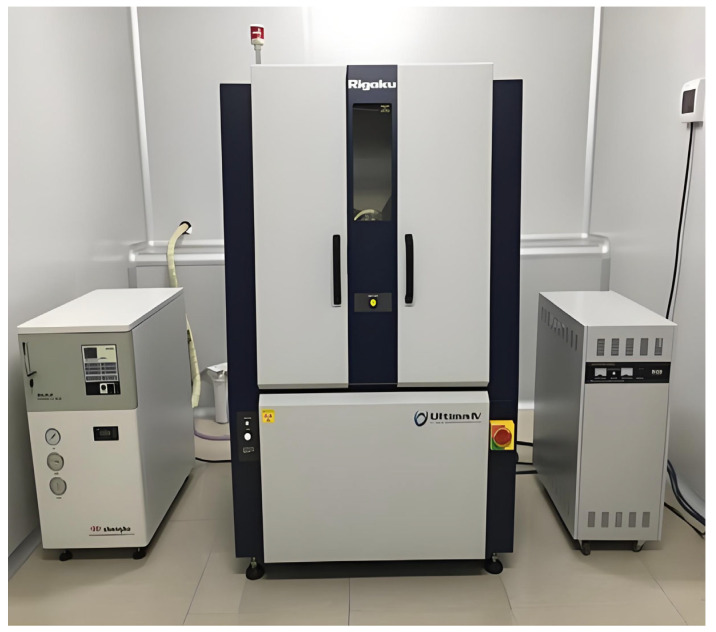
Rigaku Ultima IV X-ray diffractometer.

XRD was used to analyze the cement hydration products, the chemical composition of aeolian sand and the crystal structure of the ITZ. The instrument manufacturer was Nippon Rigaku Co, Ltd., Akishima-shi, Tokyo, Japan (Figure 4). Specimens were prepared by collecting the inner portion of hardened concrete, crushing and grinding into powder, and sieving through a 75 μm sieve. In total, 0.5 g of the powder was dried to constant weight in a vacuum drying oven, then pressed into a flat sample for testing. The test parameters were as follows: tube voltage of 40 kV, tube current of 40 mA, Cu Kα radiation (λ = 1.54 Å), 2θ range of 5–90°, and scanning speed of 5°/min. Semi-quantitative XRD analysis was performed to determine the relative content of K_2_SiO_3_ on the modified sand surface, calculated via the ratio of the integrated peak areas of the characteristic K_2_SiO_3_ diffraction peaks (near 28.5° and 30.2°) to the primary quartz peak (at 26.6°).

#### 2.3.3. Nanoindentation Test

**Figure 5:**
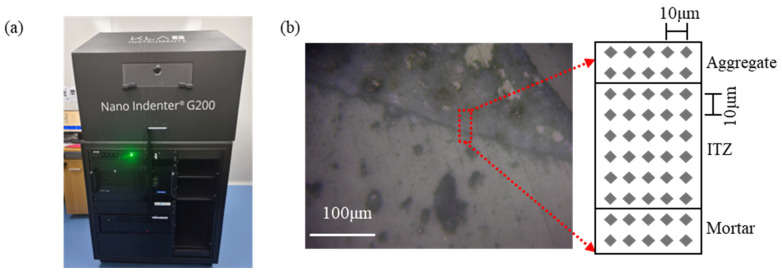
Schematic diagrams of the nanoindentation test: (**a**) Schematic diagram of the nanoindentation instrument; (**b**) Schematic diagram of the ITZ thickness test.

The thickness and the hardness of the ITZ were analyzed using a nanoindentation tester with an indentation resolution of 0.01 nm, a maximum indentation depth of 500 µm, a maximum load of 500 mN, and a load resolution of 50 nN (Figure 5a). For specimen preparation, 3 mm fragments were taken from inside the specimen for testing and immersed in anhydrous ethanol for 24 h to terminate hydration. These fragments were then dried in a drying oven at 40 °C until equilibrium weight was achieved, placed into a 25 mm × 18 mm cylindrical mold with the test side facing down, and epoxy resin was poured into the mold to cure for 24 h.

The embedded specimens were gradually polished using sandpapers with grit sizes of 180, 600, 1200, and 2000. During polishing, anhydrous ethanol was sprayed onto the sandpapers as a cooling lubricant. The polished samples were then ultrasonically cleaned with anhydrous ethanol for 3 min.

Subsequently, the sample surfaces were further polished using diamond suspensions with particle sizes of 9 μm, 3 μm, and 0.1 μm sequentially, with a total polishing time of 2 h to achieve a smooth surface. After polishing, the samples were ultrasonically cleaned with anhydrous ethanol, dried in an oven at 60 °C to constant weight, and then stored in sealed bags for subsequent tests. The nanoindentation test parameters were loading rate of 0.25 mN/s, maximum load of 2 mN, 5 × 10 indentation array with 5 μm horizontal and vertical spacing (Figure 5b).

#### 2.3.4. Fourier Transform Infrared (FTIR) Spectroscopy

FTIR is based on the principle that when the frequency of infrared light matches the vibrational frequency of molecular chemical bonds, the molecule absorbs the infrared light and undergoes vibrational-rotational energy level transitions, generating characteristic absorption peaks for molecular structure analysis. FTIR was used to detect the chemical bonds between ssDNA and the surface of aeolian sand. The Tak-CNTs sample contains the largest number of elements and can reflect the chemical bonds generated by other samples, so Tak-CNTs was selected as the object for FTIR testing.

**Figure 6:**
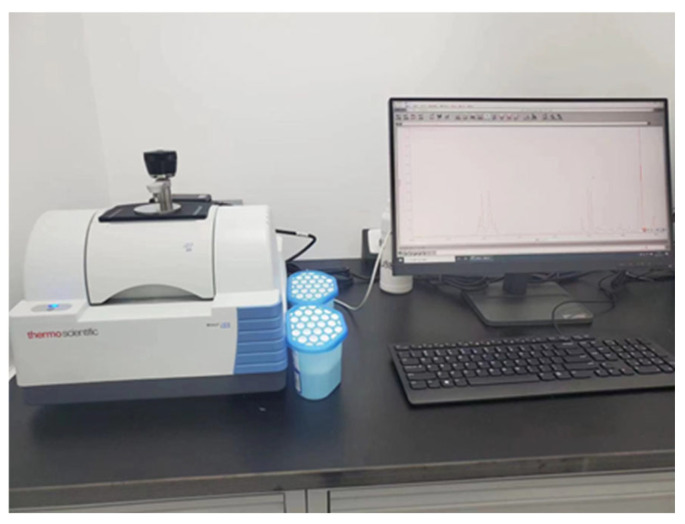
FTIR test instrument.

The test was performed using a Thermo Scientific Nicolet iS5 FTIR (Thermo Fisher Scientific, Waltham, MA, USA) (Figure 6). The test samples were prepared using the potassium bromide tableting method. The scanning range was 4000–500 cm^−1^ and the resolution was 1 cm^−1^. To obtain a clearer spectral signal, the Tak-CNTs samples were sieved through a 75 μm sieve and then used for FTIR testing; the mass ratio of the sample to potassium bromide (KBr) was set to 1:100, and the total mass of each pressed tablet was controlled at 0.2 g.

#### 2.3.5. Thermogravimetric Analysis (TGA)

**Figure 7:**
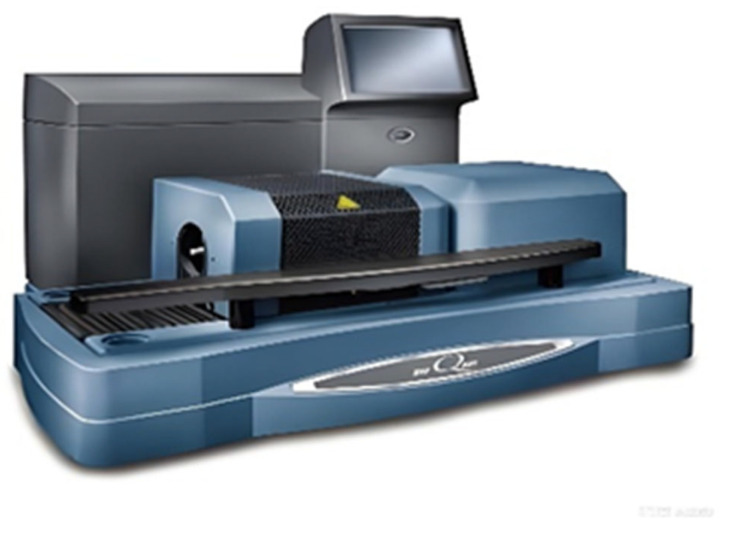
TA Q600 SDT simultaneous thermal analyzer.

TGA was used to test the type of concrete hydration products, the manufacturer of the instrument was TA, USA, the model of the equipment was Q600 SDT (TA Instruments, New Castle, DE, USA) (Figure 7), the sensitivity of the balance was 0.1 micrograms, and the range of the testing temperature was from room temperature to 1200 °C. Samples were taken from the interior of the compressive specimens to avoid the influence of surface carbonation. Particles with a volume of 1–2 cm^3^ were selected for each sample, with a total mass of approximately 10 g for testing. These particles were immersed in anhydrous ethanol for 7 d to stop hydration. After that, they were dried to constant weight at 40 °C in a vacuum drying oven. The dried samples were then crushed, ground, and sieved through a 75 μm sieve prior to TGA. In total, 0.2 g of the sample powder was weighed for testing. The samples were heated from room temperature to 1000 °C at a rate of 10 °C/min under nitrogen atmosphere. The instrument automatically records the mass loss during the heating process, and the hydration degree parameter can be calculated from the mass loss-temperature curve. The formula for calculating the degree of hydration of concrete is given by Equation (2).

(2) $$\alpha =\frac{Ldh+Ldx+0.41(Ldc)}{0.24}\times 100$$

where α is the degree of hydration and *Ldh*, *Ldx*, and *Ldc* are the weight loss in the region of dehydration (105–440 °C), dehydroxylation (440–580 °C), and decarbonation (580–1000 °C), respectively. The conversion factor for chemically bound water extracted from carbonate silicates [37] is 0.41.

#### 2.3.6. Scanning Electron Microscopy (SEM)

**Figure 8:**
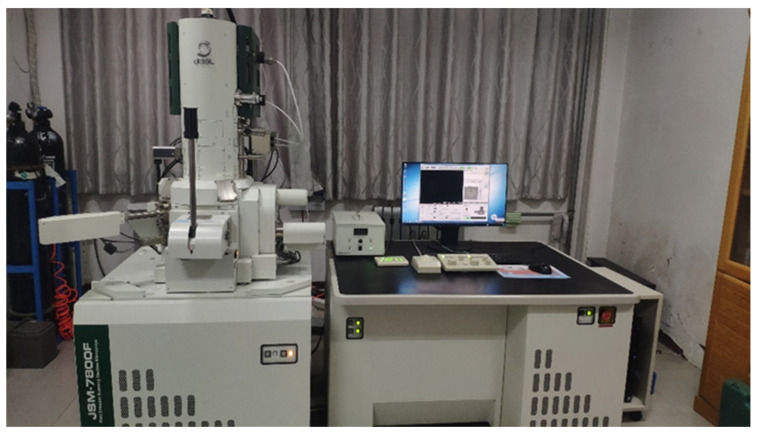
JSM-7800F scanning electron microscope.

Since the microstructure of concrete is at the micrometer level and concrete is non-conductive, SEM was selected as the observation tool; before observation, the specimens needed to be sputter-coated with gold to observe the porosity of the ITZ, the microstructure of cement hydration products, and the distribution of CNTs on the surface of aeolian sand The JSM-7800F instrument (JEOL Ltd., Tokyo, Japan) was operated at an accelerating voltage of 20 kV (Figure 8). Among them, samples used for observing the porosity of the ITZ were nanoindentation specimens. Various phases in the ITZ could be clearly distinguished under the backscattered electron (BSE) mode. Samples for observing the microstructure of hydration products were taken from the inner part of compressive specimens, with a size of approximately 1 cm^3^. One sample was randomly selected from each group for testing. For observing the dispersion of CNTs, 0.2 g of Bei-CNTs, Tak-CNTs, Kum-CNTs, and Gur-CNTs powders were randomly weighed respectively for characterization.

#### 2.3.7. Time-of-Flight Secondary Ion Mass Spectrometry (TOF-SIMS)

**Figure 9:**
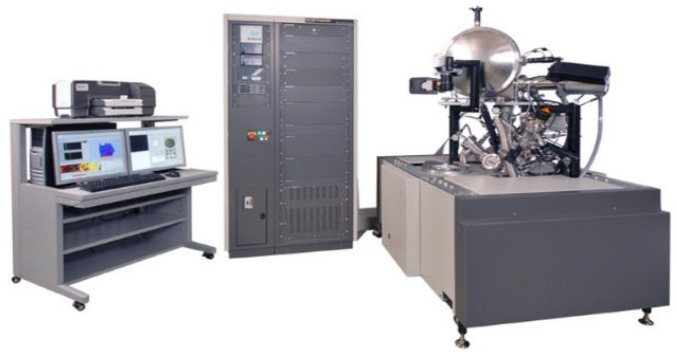
PHI NanoTOF II TOF-SIMS instrument.

TOF-SIMS was used to analyze the new compounds formed by the reaction between ssDNA and the aeolian sand surface, via mass spectrometry and mapping analysis. The chemical states and potential compounds on the sample surface were identified from the mass spectra, while the distribution of specific chemical components on the surface was determined via ion mapping. The test was performed using a PHI Nano TOF II time-of-flight secondary ion mass spectrometer manufactured by ULVAC-PHI, Inc. (ULVAC-PHI, Inc., Kanagawa, Japan) (Figure 9). A bismuth (Bi^2+^) ion source with an energy of 30 keV and an ion current of 2 nA was adopted, with a raster size of 200 × 200 µm. The mass range was set at 2–1850 u, and the high-mass-resolution mode was used.

Tak-CNTs were also selected as samples for TOF-SIMS testing. Since ssDNA/CNTs did not achieve 100% coverage on the surface of Tak sand, the Tak-CNTs samples were sieved through a 75 μm sieve to ensure the detection of the target chemical bonds. In total, 0.2 g of the sample was weighed, dried at 60 °C in a vacuum drying oven for 4 h, then wrapped with aluminum foil and sealed in a clean glass bottle until testing.

#### 2.3.8. Transmission Electron Microscopy (TEM)

**Figure 10:**
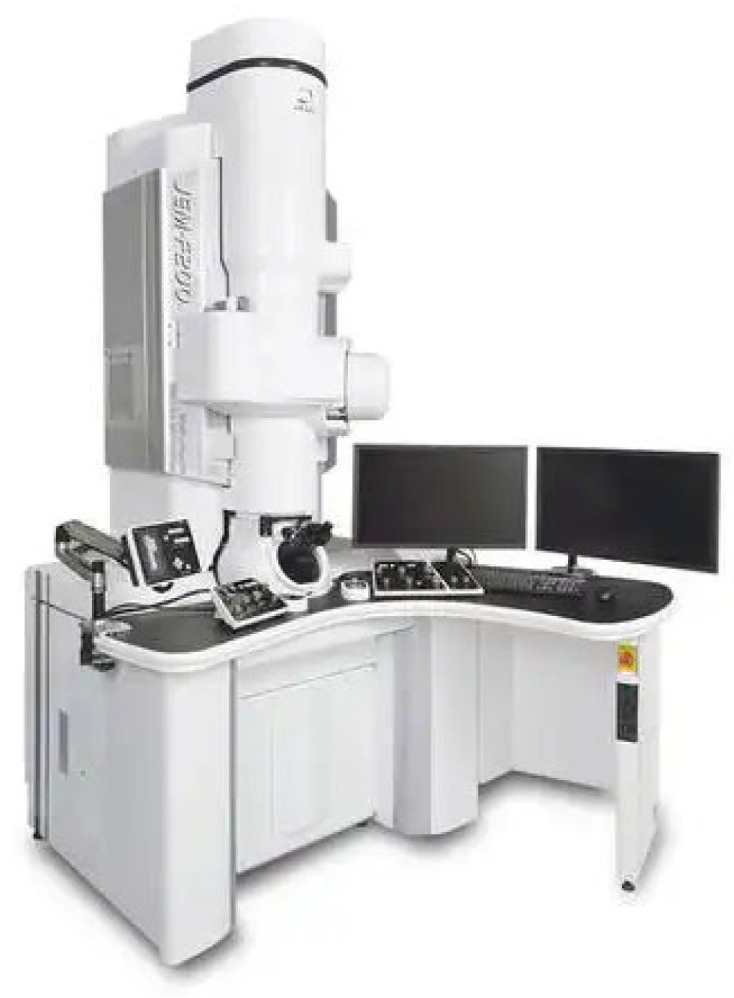
JEM-F200 transmission electron microscope.

TEM was used to analyze the crystal structure and morphology of cement hydration products. The instrument employed was a JEM-F200 transmission electron microscope manufactured by JEOL Ltd., Tokyo, Japan (Figure 10).

A 10 mL ssDNA/CNTs solution (1 mg/mL) was mixed with cement powder at a water-cement ratio (*w*/*c*) of 0.6. The mixture was ultrasonicated for 20 min using the same ultrasonic device as for CNTs dispersion, with an ultrasonic power of 500 W. To avoid overheating the cement paste, the mixture was immersed in ice water to keep the temperature below 20 °C. The supernatant of the cement paste was collected and dropped onto a 200-mesh copper grid for observation. A plain cement paste sample was prepared in the same way as a reference. The crystal morphology and interplanar spacing of hydration products were characterized to reveal the effect of ssDNA/CNTs on the microstructure and development of cement hydration products.

#### 2.3.9. Mercury Intrusion Porosimetry (MIP)

**Figure 11:**
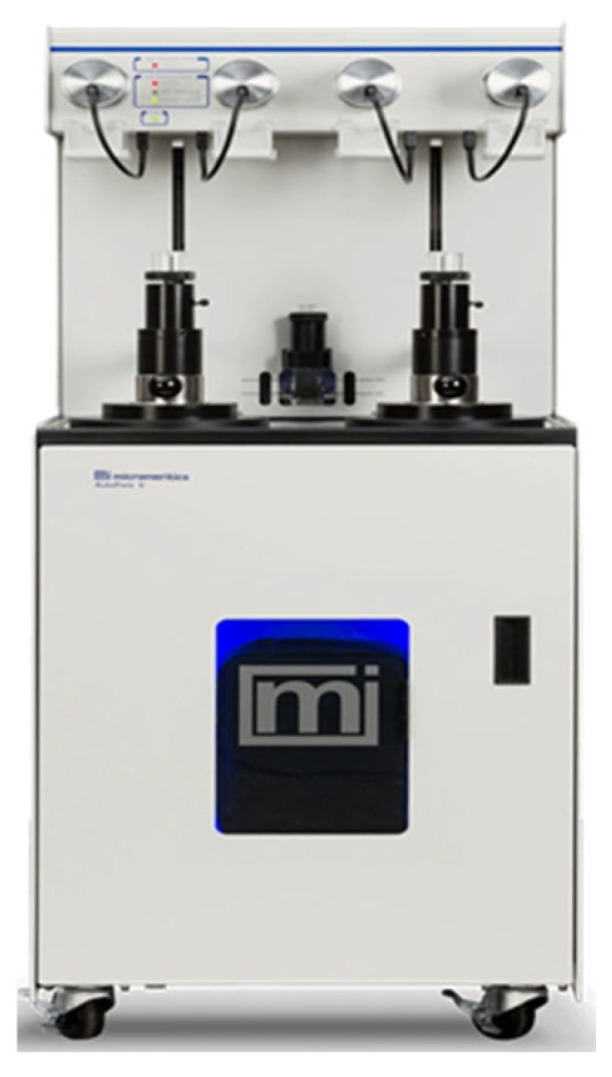
AutoPore V9500 automated mercury porosimeter.

MIP tests were performed on 8 concrete sample groups. Fragments with a volume of approximately 1 cm^3^ (weighing about 6 g each) were taken from the interior of the compressive test specimens. To terminate hydration, the fragments were immersed in anhydrous ethanol for 48 h, followed by vacuum drying at 40 °C for 24 h. The dried samples were stored in sealed bags until testing. The pore structure was characterized by using an AutoPore V9500 (Malvern Panalytical Ltd., Malvern, UK) (Figure 11) automated mercury porosimeter, with a maximum mercury injection pressure of 228 MPa, covering a continuous pore size range of 5 nm to 360 μm. From the measurements, the cumulative pore volume curves and total porosity of the samples were obtained.

#### 2.3.10. Leaching Test and pH Measurement

To evaluate the residual KOH on the modified sand surface after the triple-washing process, a leaching test was conducted. In total, 100 g of the modified aeolian sand was immersed in 100 mL of deionized water and subjected to mechanical stirring for 30 min. The pH value of the resulting supernatant was then measured using a pH meter (PHS-25, Shanghai Lichen Scientific Instrument Co., Ltd., Shanghai, China) with a precision of 0.01. This test was performed to estimate the concentration of residual free hydroxyl ions and verify the efficiency of the alkali removal.

## 3. Results and Discussion

### 3.1. Characterization of Modified Aeolian Sand

#### 3.1.1. Chemical Composition and Phase Analysis of Alkali-Modified Aeolian Sand Surfaces

**Figure 12:**
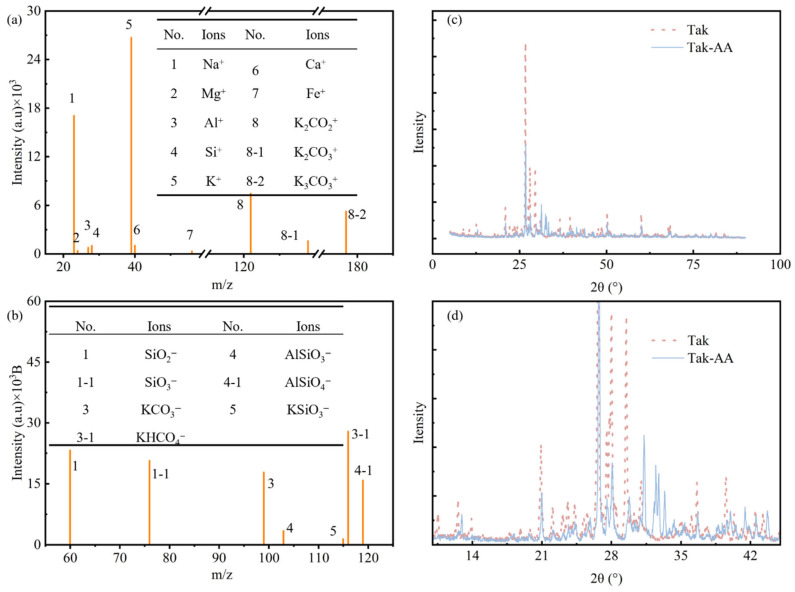
TOF-SIMS and XRD results of the KOH-modified Tak sand surface: (**a**) positive ion mass spectrum; (**b**) negative ion mass spectrum; (**c**,**d**) comparative XRD patterns of pristine Tak and Tak-AA sand.

To elucidate the modification mechanism of KOH treatment on the aeolian sand surface, a synergistic approach combining TOF-SIMS and XRD was employed. Under the action of 4 mol/L KOH, a distinct chemical transformation occurred on the sand surface. The TOF-SIMS positive ion spectrum (Figure 12a) reveals a significant enrichment of potassium signals. Combined with the negative ion spectrum (Figure 12b), it is determined that these products primarily exist as K_2_CO_3_, KHCO_3_, and K_2_SiO_3_. Furthermore, trace amounts of Mg^2+^, Fe^3+^, Ca^2+^, and Na^+^ were detected, the release of which is attributed to the alkali-induced micro-dissolution of the mineral lattice [28].

These chemical transformations were further validated by the XRD comparative analysis presented in Figure 12c,d. Compared to the pristine Tak sand, the Tak-AA sample showed several prominent new diffraction peaks. Specifically, the new peaks observed near 31.3° and 32.4° correspond to the crystalline phases of potassium carbonates, which highly align with the chemical states detected by TOF-SIMS. Meanwhile, the characteristic peaks of potassium silicate appearing near 28.5° and 30.2° provide direct evidence of the dissolution and subsequent recrystallization of surface SiO_2_ components under strong alkaline conditions

Notably, a significant decrease in the diffraction intensity of the primary minerals (e.g., the quartz peak at 26.6°) was observed in the modified sand. This phenomenon indicates that the strong alkali treatment not only induces surface dissolution and the release of active ions but also successfully constructs a layer of low-crystallinity ASL on the sand surface [38]. The ASL effectively covers the pristine crystal surface and provides high-density active sites for the subsequent chemical coordination of ssDNA. This phase-level transformation provides robust physical evidence for the “cation-mediated molecular anchoring” mechanism.

To quantitatively evaluate the residual influence of the alkali treatment, a leaching test was performed: 100 g of Tak-AA sand was stirred in 100 mL of deionized water for 30 min, and the resulting supernatant exhibited a pH of 11.90, indicating a minimal concentration of residual free hydroxyl ions. Furthermore, semi-quantitative analysis of the XRD pattern revealed that the characteristic peaks of K_2_SiO_3_ correspond to a minor fraction (approximately 1.9 wt.%) of the total mineral composition. Combined with the results of XRD semi-quantitative analysis and leaching pH test, the contents of residual KOH and potassium silicate in the aeolian sand after alkali activation in this study are far below the critical thresholds for significantly contributing to strength in alkali-activated systems. Such low-content alkaline salts cannot achieve a substantial improvement in mechanical properties through geopolymerization or salt crystallization [39,40]; thus, it can be clearly confirmed that the mechanical enhancement of the specimens is not derived from the effect of alkaline salts, but dominated by the synergistic reinforcement of the ssDNA/CNTs composite.

#### 3.1.2. Microstructural Morphology

**Figure 13:**
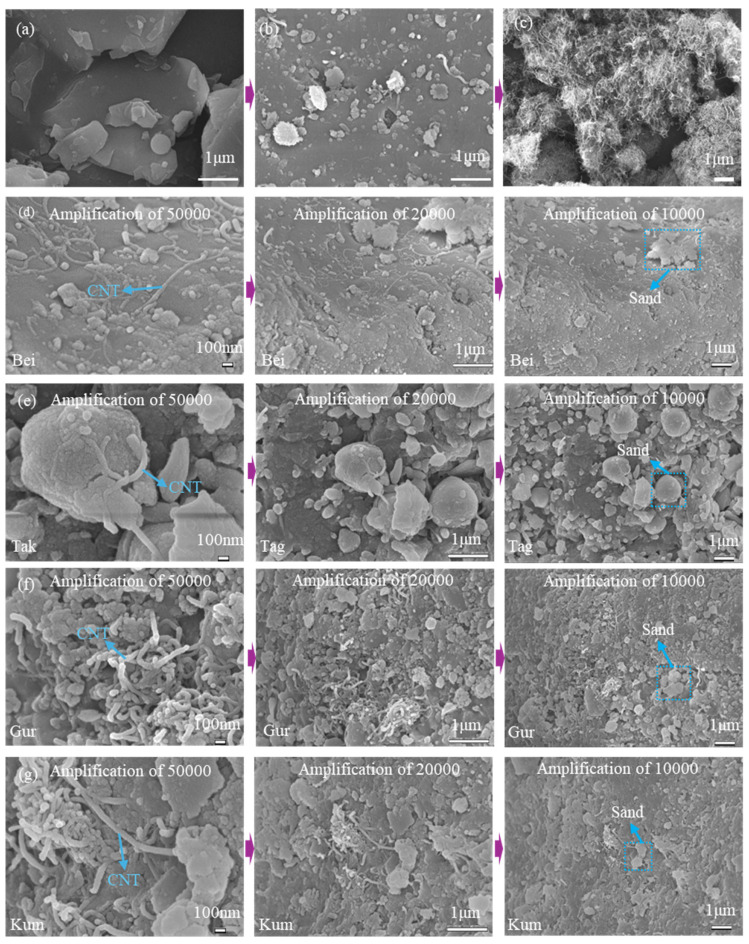
Microscopic morphology of four aeolian sands: (**a**) pristine Bei aeolian sand surface; (**b**) alkali-activated sand surface; (**c**) pristine bundled CNTs; (**d**) Bei-CNTs; (**e**) Tak-CNTs; (**f**) Gur-CNTs; (**g**) Kum-CNTs.

The microstructural evolution of the aeolian sand surface before and after modification was examined via SEM, as illustrated in Figure 13. The surface of the pristine Bei aeolian sand appears relatively smooth and chemically inert. In contrast, the alkali-activated surface (Figure 13b) displays a significantly roughened morphology with numerous protruding white particles, providing direct visual evidence of the surface micro-dissolution and etching effects induced by the 4 mol/L KOH solution. This process disrupts the stable mineral structure to form the ASL rich in active cationic sites.

Regarding the reinforcement phase, the pristine CNTs (Figure 13c) exist as dense, micron-scale agglomerated bundles due to strong inter-tube van der Waals forces. Following the ssDNA-assisted modification, a substantial improvement in the dispersion state is observed across all four types of aeolian sand (Figure 13d–g). While slight agglomeration remains visible—a known limitation of current ultrasonic dispersion technologies that prevents the total separation of all bundles into isolated nanotubes—the morphology is markedly enhanced compared to the pristine bundles. Notably, a high density of individual, dispersed CNTs is successfully anchored to the ASL, acting as “nano-bridges” at the interface.

#### 3.1.3. Molecular Structure and Chemical Bonding

**Figure 14:**
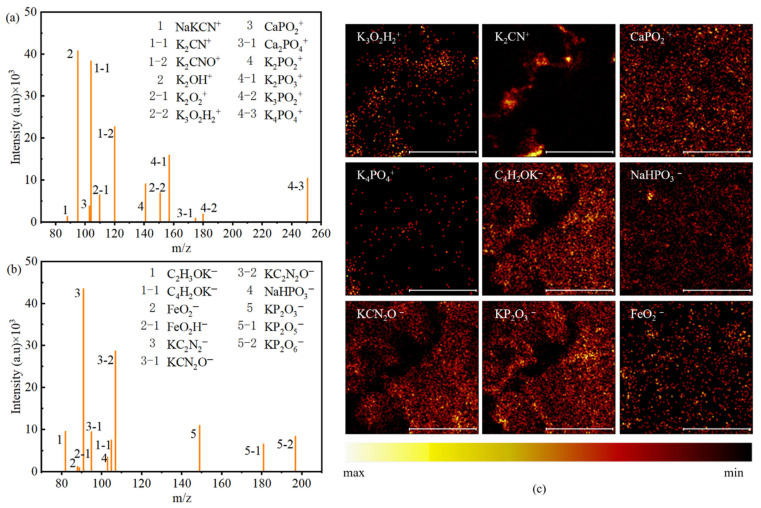
TOF-SIMS results of CNTs anchored on the surface of Gur. (**a**) Positive ion mass spectrum; (**b**) negative ion mass spectrum; (**c**) element distribution imaging of positive and negative ions on the surface of Gur, scale bar: 100 μm.

TOF-SIMS was employed to detect the chemical bonds formed between ssDNA and the aeolian sand surface. Since all four aeolian sands were modified with KOH solution, the primary cations present were potassium and calcium ions. The Tak sample was selected for detailed characterization, as it contains all the key mineral phases present in the other three sands, with the results shown in Figure 14. The positive ion spectrum (Figure 14a) and negative ion spectrum (Figure 14b) confirm that potassium and sodium ions on the sand surface form chemical bonds with the nitrogen-containing groups of ssDNA, while iron ions chelate with carbonyl groups. The presence of phosphate groups is also evident from both spectra, indicating their chemical bonding with cations such as potassium, sodium, and calcium. This is consistent with findings from a previous study [41]. The ion distribution diagrams in Figure 14c confirm that ssDNA is uniformly distributed on the aeolian sand surface, primarily through phosphate groups bonded with these cations.

**Figure 15:**
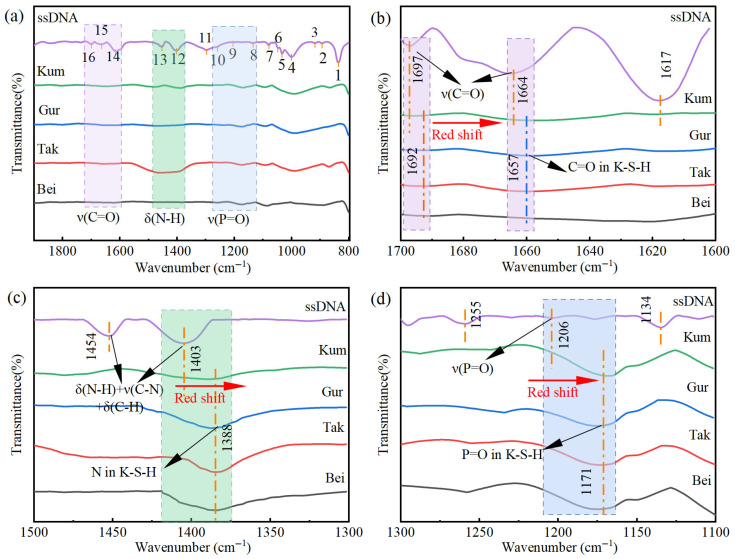
Wavenumber shifts in the FTIR spectra of the four modified aeolian sands. (**a**) overall FTIR spectra in the wavenumber range of 800–1800 cm^−1^; (**b**) red shifts of carbonyl peaks at 1697 cm^−1^ and 1664 cm^−1^ (**c**) shift of nitrogenous base peak from 1403 cm^−1^ to 1388 cm^−1^; and (**d**) characteristic peak of phosphate groups at 1206 cm^−1^.

The analysis of vibrational peak shifts in FTIR spectra, illustrated in Figure 15 and Table 4, characterizes the chemical bonding of ssDNA/CNTs on aeolian sand surfaces within the 800–1800 cm^−1^ range [41]. Red shifts in carbonyl peaks from 1697 cm^−1^ and 1664 cm^−1^ (shifts of 5 cm^−1^ and 7 cm^−1^) indicate minor participation in chemical bonding, given their low prominence in modified sand spectra (Figure 15b). Similarly, nitrogenous base shifts from 1403 cm^−1^ to 1388 cm^−1^ confirm the involvement of amino groups and ring nitrogen atoms (Figure 15c).

The most pronounced evidence of bonding is the distinct band at 1206 cm^−1^ (Figure 15d), where significantly larger peak areas for modified sands align with TOF-SIMS results to demonstrate primary phosphate group involvement. Consequently, the chemical connection is predominantly driven by phosphate group coordination with surface cations, while nitrogenous bases and carbonyl groups remain secondary contributors to the modification.

### 3.2. Mechanical Properties

**Figure 16:**
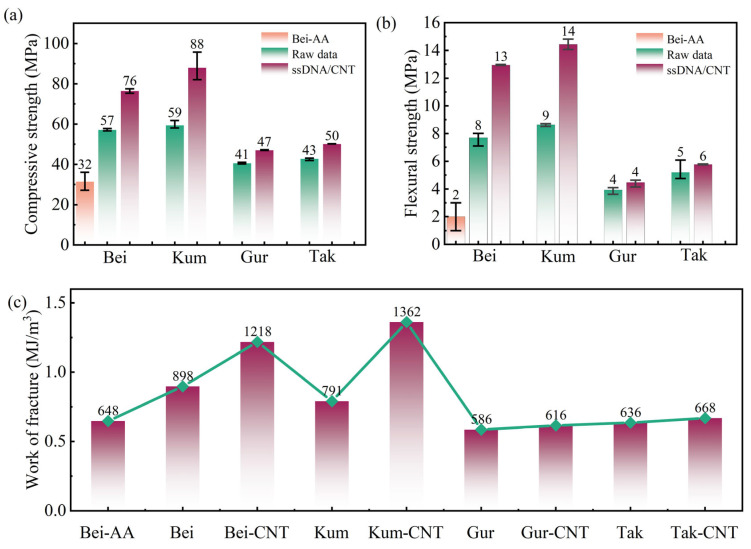
Mechanical properties of aeolian sand concrete: (**a**) compressive strength; (**b**) flexural strength; (**c**) compressive energy dissipation.

Figure 16 presents the stress–strain curves, compressive and flexural strengths of the four aeolian sands concrete after ssDNA/CNTs modification. Relative to the unmodified control groups, the modified concretes exhibited increases in compressive strength of 34%, 48%, 16%, and 17% for the Bei, Tak, Gur, and Kum groups, respectively (Figure 16a). The corresponding improvements in flexural strength were 68%, 67%, 14%, and 11%, respectively (Figure 16b). Additionally, the compressive strain and flexural deflection were significantly enhanced for all modified groups (Figure 16a,b). The compressive energy dissipation of Kum-CNTs and Bei-CNTs increased by 72% and 36% compared to the unmodified Kum and Bei groups, respectively, while the enhancements for Gur-CNTs and Tak-CNTs were also substantial (Figure 16c). Notably, the flexural stress-displacement curves of the four aeolian sands exhibited a stepwise increasing trend, further indicating that ssDNA/CNTs enhanced the stress dissipation effect and crack bridging capacity of aeolian sand concrete.

As the control group, Bei-AA specimens (containing only ASL without anchored ssDNA/CNTs) exhibited significantly lower performance than Bei-CNTs: compressive strength decreased by 104%, flexural strength dropped by 105%, and compressive and flexural strain capacities were reduced by 67% and 119%, respectively. This comparison confirms that the synergistic effect of ssDNA and CNTs is the core mechanism underlying the improvement in concrete performance. Their roles include (a) CNTs act as stress-transfer carriers to enhance ITZ strength; (b) DNA molecular chains optimize pore structure through hydrogen-bond networks; and (c) together they form a composite reinforcement system of “nano-enhancement–molecular bridging.”

### 3.3. Pore Structure

**Figure 17:**
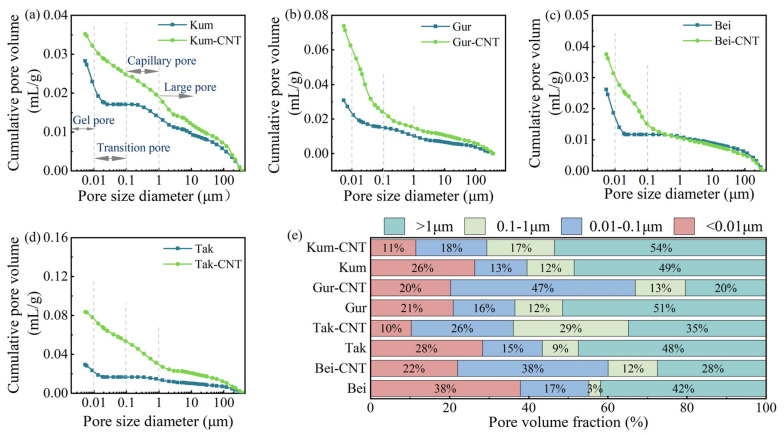
Pore size distribution of the four aeolian sand concretes: (**a**) cumulative pore volume curves of Kum and Kum-CNTs; (**b**) cumulative pore volume curves of Gur and Gur-CNTs; (**c**) cumulative pore volume curves of Bei and Bei-CNTs; (**d**) cumulative pore volume curves of Tak and Tak-CNTs; (**e**) volume fraction of pores with different sizes.

MIP was conducted to characterize the nanoscale to microscale pore structure of the aeolian sand concrete, with results presented in Figure 17. Based on Hodot’s pore classification method [42], the pores were categorized into gel pores (<0.01 μm), transition pores (0.01–0.1 μm), capillary pores (0.1–1 μm), and macropores (>1 μm). As shown in Figure 17a–d, the cumulative pore volumes of the four modified concretes (Kum-CNTs, Gur-CNTs, Bei-CNTs, and Tak-CNTs) were 0.035 mL/g, 0.073 mL/g, 0.037 mL/g, and 0.083 mL/g, respectively. In contrast, the cumulative pore volumes of the pristine aeolian sand concretes (Kum, Gur, Bei, Tak) were 0.028 mL/g, 0.031 mL/g, 0.026 mL/g, and 0.029 mL/g.

Although the total cumulative pore volume increased after CNTs modification, a profound redistribution of pore sizes occurred, following a clear “pore refinement” mechanism. According to the literature, pores larger than 1 μm are considered hazardous to the structural integrity of concrete [43]. The experimental data in Figure 17e reveals that despite the rise in total porosity, the percentage of these harmful macropores was dramatically reduced in the modified specimens. This reduction in harmful porosity demonstrated a positive correlation with sand particle size, with the exception of the Kum-CNTs concrete, where the proportion of harmful pores increased. This phenomenon is attributed to the presence of large-sized flake particles inherent to Kum sand, as detailed in Section 3.1.1.

The overall results indicate that the synergistic effect of the ASL and the ssDNA/CNTs nanocomposite effectively facilitates the conversion of large, harmful pores into beneficial gel and transition pores. This optimization of the pore structure, achieved through the molecular-scale anchoring of CNTs, significantly enhances overall performance metrics such as strength and energy dissipation capacity, despite the moderate increase in total porosity.

### 3.4. Hydration Products and Their Microstructure

#### 3.4.1. Effects of ssDNA/CNTs on Crack Morphology

**Figure 18:**
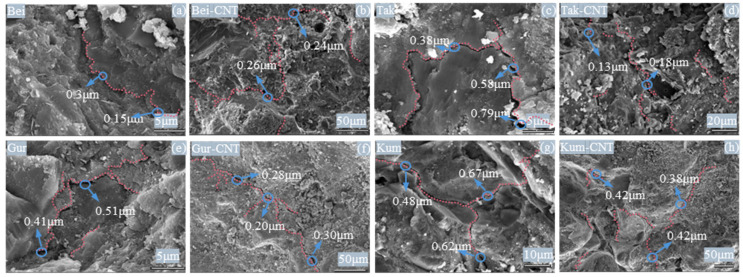
Representative SEM images of aeolian sand concrete. (**a**) Bei; (**b**) Bei-CNTs; (**c**) Tak; (**d**) Tak-CNTs; (**e**) Gur; (**f**) Gur-CNTs; (**g**) Tak; (**h**) Tak-CNTs. The red dashed lines indicate microcracks in the concrete.

SEM imaging was used to elucidate the effect of CNT-modified aeolian sand on the morphology of the concrete matrix. As shown in Figure 18a,c,e,g, unmodified aeolian sand concrete exhibits a single main crack with a width ranging from 0.3 to 0.7 μm and smooth crack edges. In contrast, the CNT-modified concrete (Figure 18b,d,f,h) shows a more complex fracture pattern. In addition to one main crack, many microcrack bifurcations are generated in the matrix, resembling a tree trunk-branches structure. Furthermore, the widths of the main cracks in the modified matrix are generally less than 0.3 μm, with rougher crack edges. These observations indicate that well-dispersed CNTs can effectively control microcrack propagation due to their high aspect ratio and large specific surface area.

#### 3.4.2. Effects of ssDNA/CNTs on C-S-H Crystal Structure

**Figure 19:**
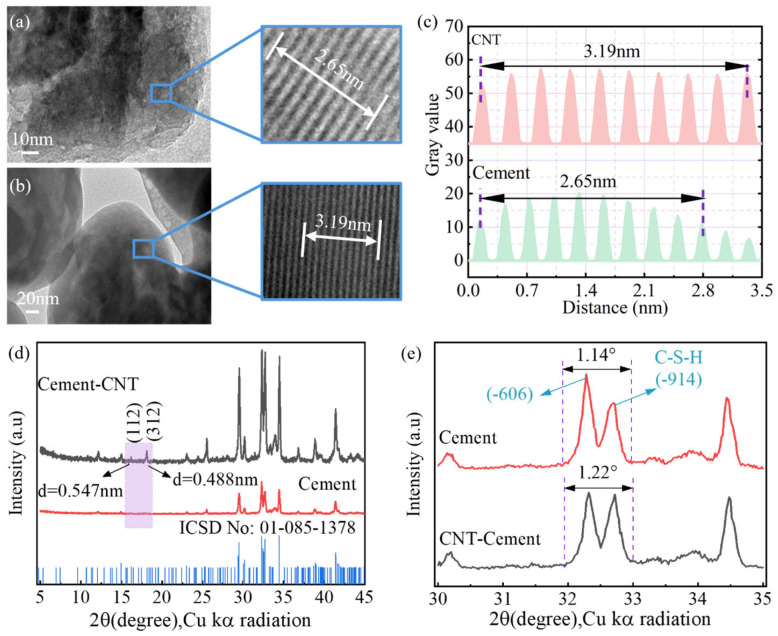
TEM and XRD analysis of cement hydration products: (**a**) high-resolution TEM image of plain cement paste; (**b**) high-resolution TEM image of ssDNA/CNT-modified cement paste; (**c**) IFFT results of plain cement paste and modified cement paste; (**d**) XRD patterns of plain cement paste, modified cement paste, and the standard C-S-H card; (**e**) Characteristic XRD peaks of plain cement paste and modified cement paste.

The incorporation of ssDNA/CNTs into the hydration process induces a fundamental transformation in the C-S-H crystal structure. As illustrated in Figure 19a–c, the pristine cement paste exhibits a conventional C-S-H laminar morphology with a characteristic d-spacing of approximately 0.265 nm [44,45]. In contrast, the presence of ssDNA/CNTs results in a more ordered stacking of C-S-H layers, with an expanded d-spacing of 0.319 nm. This structural shift originates from the rotation or translation of neighboring crystal faces, driven by the higher Ca/Si ratio and resultant silicate chain defects induced by the biomolecules [46]. Furthermore, the binding of Ca^2+^ ions to ssDNA chains distorts the calcium polyhedral layers, increasing interplanar distances.

This ordered stacking is corroborated by XRD analysis (Figure 19d,e), which reveals new crystalline facets at (112) and (312) and an increase in the full width at half maximum (FWHM) of the characteristic C-S-H peak from 1.14° to 1.22°, signifying a higher degree of disorder among calcium atoms within the facets. Collectively, the abundant functional groups of ssDNA facilitate robust bio-inorganic chemical connections, transforming the C-S-H into a dense, locally reinforced composite. This ordered stacking increases the effective contact area during compaction, enhancing chemical bonding and interfacial interaction, which ultimately underpins significant improvements in the macroscopic mechanical properties of the aeolian sand concrete.

#### 3.4.3. Effects of ssDNA/CNTs on Hydration Products and Hydration Degree

**Figure 20:**
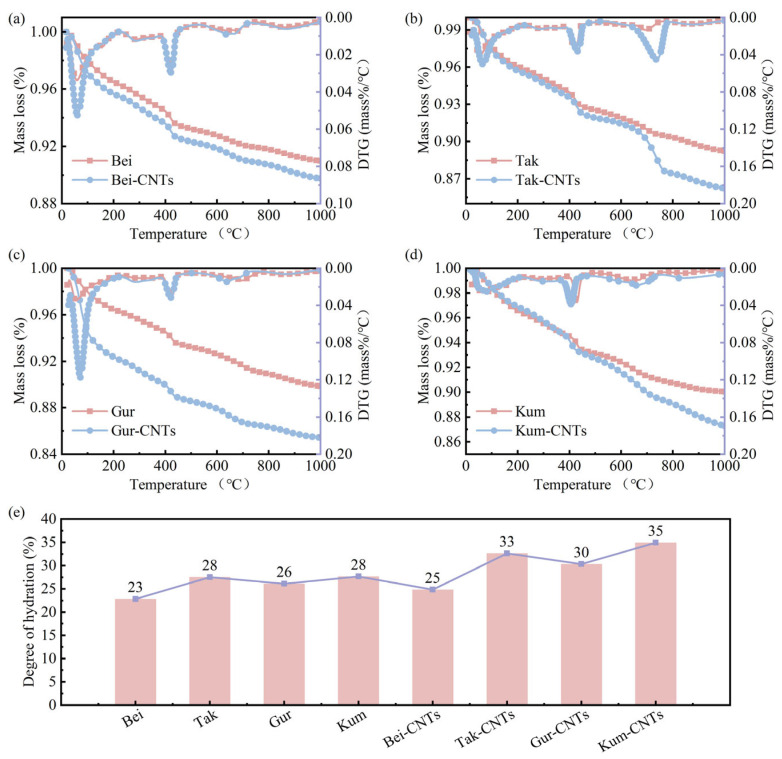
Hydration product analysis and hydration degree calculation: (**a**–**d**) TGA/DTG curves of eight samples. (**e**) Hydration degree of eight samples.

TGA-DTG curves were used to analyze the effect of ssDNA/CNTs on the hydration products and degree of hydration in aeolian sand concrete. Figure 20a–d presents the DTG-TGA curves for eight samples. The three main peaks in the DTG curves correspond to the dehydration of C-S-H (around 100 °C), dehydroxylation of silicates (around 440 °C), and decarbonation of carbonates (around 720 °C). Additionally, there are peaks corresponding to the dehydration of ettringite (AFt) and monosulfide-type hydration products (AFm) around 100 °C. Figure 20e shows the degree of hydration for different samples. It is evident that the hydration degree of ssDNA/CNT-modified concretes show varying enhancements, with Kum-CNTs exhibiting the largest enhancement of 25%. This result indicates that the incorporation of ssDNA/CNTs positively promotes the cement hydration process, which in turn contributes to the improved mechanical properties and durability of the concrete.

### 3.5. Concrete Interface Transition Zone

#### 3.5.1. Effects of ssDNA/CNTs on ITZ Thickness

**Figure 21:**
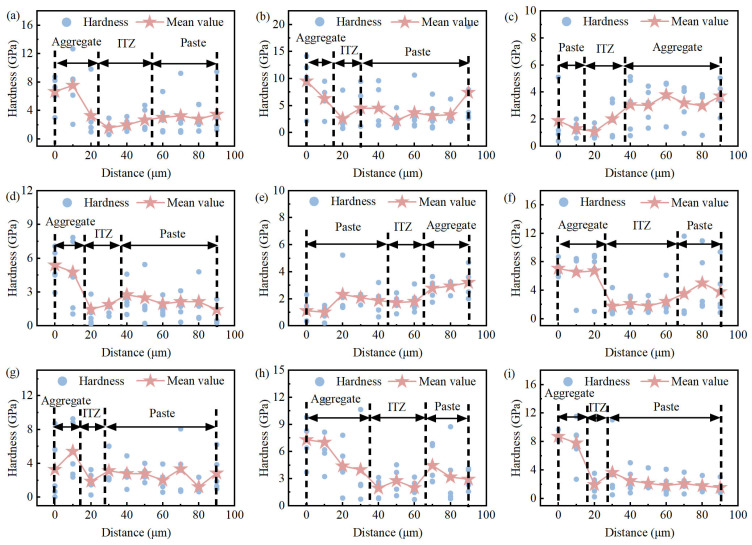
Distribution of hardness in the aggregate surroundings. (**a**) Bei; (**b**) Bei-CNTs; (**c**) Bei-AA; (**d**) Gur; (**e**) Gur-CNTs; (**f**) Kum; (**g**) Kum-CNTs; (**h**) Tak; (**i**) Tak-CNTs.

In concrete, the ITZ is a special region between cement paste and aggregate, whose thickness and properties have a crucial impact on the overall mechanical properties and durability of concrete. To accurately determine the thickness of the ITZ in aeolian sand concrete, this study used nanoindentation to map the hardness distribution across the aggregate-paste interface (Figure 21). The ITZ thickness was defined based on the hardness variation characteristics: starting from the aggregate edge, the region where the hardness decreases sharply relative to the aggregate, then begins to recover and gradually approaches the hardness of the bulk cement paste, was identified as the ITZ, with the corresponding distance range defined as the ITZ thickness.

Figure 21 presents the hardness distribution of the ITZ. Based on the above definition, the results showed that the ITZ thickness of ssDNA/CNTs modified concrete ranged between 10–15 μm, whereas the ITZ thickness of the unmodified concrete ranged between 20–40 μm. This comparison clearly indicates that the incorporation of ssDNA/CNTs significantly reduced the ITZ thickness of aeolian sand concrete. In contrast, the ITZ width of Bei-AA was about 30 μm, which was reduced by 25% relative to the 40 μm of Bei.

#### 3.5.2. Effects of ssDNA/CNTs on ITZ Porosity

**Figure 22:**
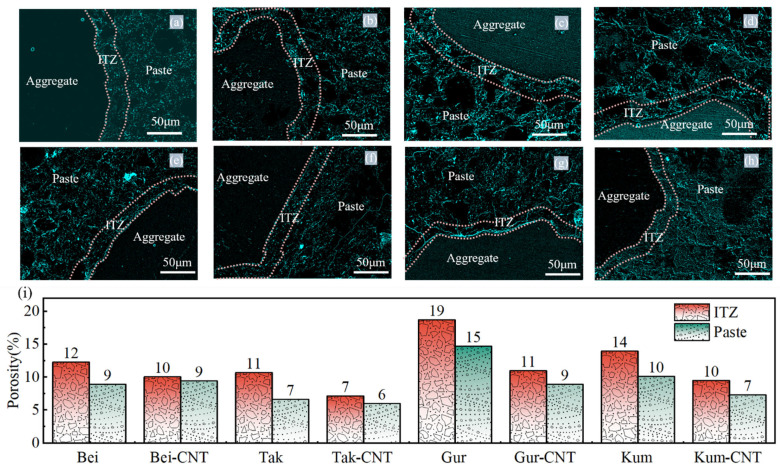
BSE image analysis of ITZ porosity: (**a**–**h**) binarized images corresponding to the BSE maps; (**i**) ITZ and bulk paste porosity of the eight sample groups.

The influence of ssDNA/CNT-modified aeolian sand on the porosity of the concrete ITZ was quantitatively evaluated using BSE imaging. As illustrated in Figure 22a–h, the distinct physical phases within the concrete matrix were accurately differentiated based on gray-level contrast, where pores and cracks are represented by black regions [47,48]. In the unmodified aeolian sand concretes, a significant disparity in porosity between the ITZ and the paste was observed, with differences ranging from 21.6% to 37.9%. This phenomenon is primarily attributed to the “wall effect” at the aggregate interface, which leads to a loose packing of cement particles and inherently higher porosity in the ITZ compared to the bulk paste.

Following modification with the ASL and ssDNA/CNTs, the porosity disparity was substantially narrowed. For the modified specimens (Bei-CNTs, Tak-CNTs, Gur-CNTs, and Kum-CNTs), the differences between ITZ and paste porosity decreased to 6.4%, 16.6%, 18.9%, and 23.0%, respectively. Notably, the Tak-CNTs specimen exhibited a remarkable 63% reduction in ITZ porosity. This refinement of the ITZ microstructure is consistent with the “pore refinement” mechanism discussed in Section 3.3. The formation of a flexible ASL–ssDNA/CNTs–C-S-H polymer network effectively fills and densifies the interfacial voids. By reducing stress concentration and restricting the channels for the penetration of harmful substances, this modification strategy significantly strengthens the weak interfacial link, thereby enhancing the overall mechanical integrity and durability of the aeolian sand concrete.

## 4. Discussion

### 4.1. Coupling Effect Between the ASL of Aeolian Sand and Cement Hydration

Based on the TOF-SIMS results, a large number of soluble salts were generated on the surface of aeolian sand, forming an active layer containing K^+^, Ca^2+^, Fe^3+^and Na^+^ ions. During the cement hydration process, this active layer releases a significant amount of K^+^, Ca^2+^, and Na^+^ ions (as shown in Equation (3)), with the hydrolysis process of Ca^2+^ and Na^+^ being like that of K^+^. These ions can participate in the cement hydration reaction to form AFt [49], as shown in Equation (4).

**Figure 23:**
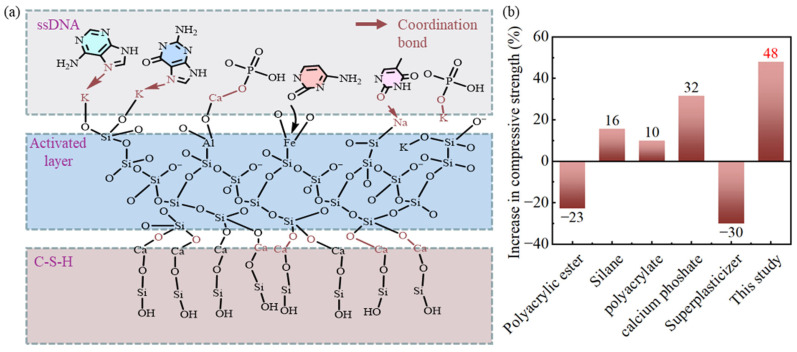
The formation of an active layer on the surface of alkali-modified aeolian sand and its chemical bonding with ssDNA: (**a**) binding mechanism of active layer with CSH and ssDNA; (**b**) comparison of the compressive strength enhancement rate of concrete with different aggregate surface modification methods.

Additionally, in accordance with the research of Rossignol et al. [38], the silicate ions in the active layer are connected to the substrate via Si-O-Si bonds, which are much stronger than the hydrogen bond connections between existing hydration products and aggregates (as shown in Figure 23). Simultaneously, the active layer undergoes chelation reactions with phosphate, carbonyl, and amine groups on the ssDNA chain, firmly anchoring the ssDNA/CNTs on the surface of aeolian sand [38]. This ensures good dispersion of CNTs in the concrete matrix, as confirmed by the TOF-SIMS and FTIR results.

(3) $$K_{2}{\mathrm{SiO}}_{3}{+2H}_{2}O\to {2K}^{+}{+H}_{2}{\mathrm{SiO}}_{3}{+2\mathrm{OH}}^{-}$$

(4) $$\begin{array}{c} x{\mathrm{CaO}+\mathrm{ySiO}}_{2}{+\mathrm{nH}}_{2}O\to x\mathrm{CaO}\cdot {\mathrm{ySiO}}_{2}\cdot nH_{2}O\\ {2[\mathrm{Al}(\mathrm{OH})}_{4}{]}^{-}{+3\mathrm{SO}}_{4}^{2-}{+6\mathrm{Ca}}^{2+}{+4\mathrm{OH}}^{-}{+26H}_{2}O\to C_{3}A\cdot {3\mathrm{CaSO}}_{4}\cdot {32H}_{2}O \end{array}$$

As illustrated in Figure 23b, the proposed ASL–ssDNA/CNTs modification strategy demonstrates superior performance compared to conventional aggregate treatment methods. Previous studies using polymer-anchored CNTs or superplasticizer-dispersed CNTs frequently reported compressive strength reductions of up to 23% [19,50] or only marginal gains [51]. Alternative treatments using silane [20] and calcium phosphate [52] have shown effectiveness by promoting chemical bonding between C-S-H and the aggregate, but the novel method proposed in this study achieves the highest enhancement rate by coupling surface hydrolysis with hydration reactions.

The ASL facilitates this through two primary synergistic mechanisms: first, the release of Ca^2+^ from the activated surface promotes further cement hydration and the formation of AFt, which effectively fills microcracks and pores within the ITZ. This densification is corroborated by the increased hardness and modulus observed in the nanoindentation results (Section 3.5.1) and the significant reduction in ITZ porosity.

Second, the ASL serves as a robust chemical bridge where abundant metal ions interact with the phosphate and carbonyl groups of ssDNA via chelation and ionic bonding [27]. This molecular-scale bio-inorganic coupling fundamentally transforms the interface from a simple physical bond to a reinforced chemical phase. The efficacy of this ITZ optimization is further evidenced by the significant narrowing of the ITZ thickness (Section 3.5.2), which collectively underpin the superior mechanical integrity and durability of the modified aeolian sand concrete.

### 4.2. Strengthening and Toughening Mechanism of ssDNA/CNTs Interaction with C-S-H

**Figure 24:**
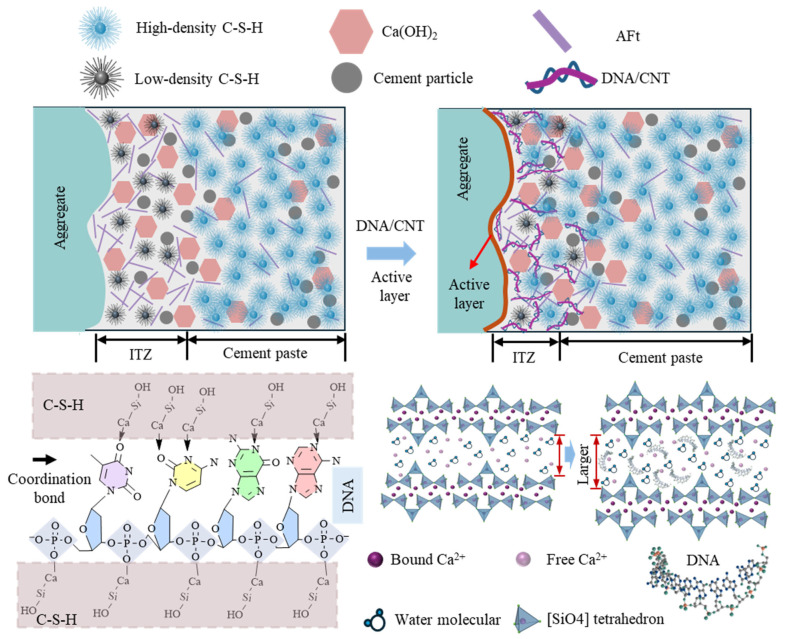
Physical model of ITZ in ssDNA/CNT-modified aeolian sand concrete.

Based on the multi-scale characterization of the ITZ and macroscopic properties, a physical model is proposed in Figure 24 to elucidate the reinforcement mechanism of the modified aeolian sand concrete. During cement hydration, ssDNA actively participates in the process by leveraging its unique chemical structure to bind with hydration products, gradually forming a flexible ASL–ssDNA/CNTs–C-S-H polymer network. The ASL provides high-density reaction sites that promote product growth, while the phosphate groups of ssDNA facilitate ionic bonding and chelation with the cement matrix. Within this network, CNTs function as high-modulus nano-reinforcements, providing crucial bridging effects that enhance overall toughness and structural efficiency of the concrete.

Although the ITZ remains a region of relatively lower density compared to the bulk paste due to the inherent “wall effect” (Section 3.5.2), the modification significantly mitigates these defects. As shown in Figure 22i, the ITZ porosity of modified specimens decreases substantially, with a remarkable 63% reduction observed in the Tak specimens. This flexible polymer network effectively fills and refines ITZ voids; for instance, the proportion of harmful pores in Bei specimens decreased from 79% to 62%, and the ITZ width narrowed from 20–30 μm to 10–15 μm (a maximum reduction of 67%). Ultimately, the synergistic effect of the active layer and ssDNA/CNTs facilitates the formation of high-density C-S-H gel and the bridging of nanoscale cracks. This denser packing structure and enriched chemical bonding enhance the interfacial energy dissipation mechanism, thereby significantly improving the peak strength, deformation performance, and overall toughness of the aeolian sand concrete.

### 4.3. Cost and Carbon Emission Analysis

The analysis results of raw material costs and carbon emissions involved in this study are detailed in Table 5. Given that carbon emissions and raw material costs are significantly influenced by multiple factors such as geographical distribution and production processes, and there is currently no unified standard data, the data in Table 5 are systematically organized based on public information.

The performance of the concrete prepared in this study is close to the C80 strength grade, so C80 concrete is used as the benchmark for comparative analysis of cost and carbon emissions. Due to the lack of carbon emission data for ssDNA raw materials, their usage accounts for a very small proportion. For the evaluation, carbon nanotube emission data were employed as a proxy for the calculation. The cost and carbon emissions of ordinary C80 concrete are precisely calculated based on the C80 concrete mix proportion specifications in JGJ 55-2011 [53]. It should be noted that the cost and carbon emission data for aeolian sand only consider the material’s intrinsic properties, so their cost and carbon emission values are both calculated as 0. The comparison results show that the concrete prepared in this study has a 63.9% increase in cost and a 294.7% increase in carbon emissions compared to ordinary C80 concrete.

Table 6 presents a comparison of the costs and carbon dioxide emission equivalents between this study and the traditional nanomodification method. The data selected for comparison is sourced from reference [14]. It can be observed that the cost of the graphene oxide (GO) modification method is 200 times that of this study, and its carbon dioxide emission equivalent is 1.28 times that of this study. This demonstrates significant advantages of this study in terms of cost and environmental impact.

In terms of scalability, this study is influenced by the fineness modulus of sand, whereas GO modification is not affected by the intrinsic properties of sand and can be applied to all types of sand. This study utilizes KOH, which generates waste alkaline solutions, while the GO modification employs reagents such as ethanol and hydrochloric acid, producing waste acids, organic substances, and other environmentally impactful wastes. Additionally, the GO modification process requires prolonged, multi-step high-temperature drying, resulting in high energy consumption.

Considering both economic efficiency and the preparation process, the advantages of this study in terms of cost and environmental impact will be further amplified during large-scale production, enabling enterprises to achieve higher economic and social benefits.

## 5. Conclusions

This study investigated the reinforcement mechanism of aeolian sand concrete modified by ASL and ssDNA-dispersed CNTs. Based on the experimental results and multi-scale characterization, the following conclusions can be drawn:

(1) Chemical anchoring mechanism: Treatment with 4 mol/L KOH successfully induces micro-dissolution of aeolian sand surface, constructing an ASL rich in active ion sites (Ca^2+^, K^+^, Na^+^, and Fe^3+^). A subsequent triple-washing process effectively removes excess unreacted alkali and soluble silicates, resulting in a low residual pH of 11.90 and minimal K_2_SiO_3_ content (~1.9 wt.%). This ensures that the precise anchoring of CNTs via ssDNA functional groups transforms the aggregate interface from physical adhesion to robust chemical coupling rather than simple salt crystallization.

(2) Macroscopic mechanical enhancement: Compared to pristine aeolian sand concrete, the synergistic modification results in substantial performance gains, with increases of up to 48%, 67%, and 42% in compressive strength, flexural strength, and compressive energy dissipation, respectively. In contrast, concrete treated only with alkali (AA-group) showed significant strength degradation, highlighting the critical role of the ssDNA/CNTs reinforcement phase.

(3) ITZ microstructure refinement: The modification leads to a significant densification of the interfacial transition zone. The ITZ thickness was reduced from 20–40 μm to 10–15 μm (a maximum reduction of 67%), and the ITZ hardness increased by 35.7%. This refinement effectively bridges the gap between the bulk paste and the aggregate, reducing stress concentration at the interface.

(4) Pore structure optimization: A clear “pore refinement” mechanism was identified via MIP analysis. Despite a moderate increase in total porosity, the proportion of harmful macropores (>1 μm) was dramatically reduced from 51% to 20%. This redistribution towards beneficial gel and transition pores significantly enhances the overall structural integrity and durability potential of the concrete.

(5) Hydration and crystallographic transformation: The presence of the ASL–ssDNA/CNT system increased the cement hydration degree by up to 25%. TEM analysis confirmed that the modification altered the crystalline stacking of hydration products, expanding the d-spacing of C-S-H crystals from 0.265 nm to 0.319 nm, resulting in a more optimized and denser microstructure.

(6) Sustainability and future perspectives: While achieving high performance through the ASL–ssDNA/CNT system, the current strategy results in increased costs and carbon emissions. Furthermore, although the concentrations of residual KOH and potassium silicate are extremely low due to the washing process, the long-term stability and degradation mechanisms of ssDNA-mediated chemical bonds in diverse and complex environmental conditions (e.g., acid rain, freeze–thaw cycles) must be prioritized in future research to ensure the long-term reliability of this bio-inorganic composite.

## Figures and Tables

**Table 1:** Main mineral components of the aeolian sands (%).

Component		Category			
Common Name	Chemical Formula	Bei	Tak	Gur	Kum
Chlorite	Mg_5.0_Al_0.75_Cr_0.23_Al_0.95_Si_3.04_O_10_(OH)_8_		5.8		
Microcline	K(AlSi_3_O_8_)	14.5	14.2	14.6	14.4
Kaolinite	Al_4_(OH)_8_(Si_4_O_10_)	3	1	6.6	4.2
Albite	Na(AlSi_3_O_8_)	29.5	24.9	36.6	36.5
Quartz low	SiO_2_	51.4	27.8	42.3	38
Calcite	Ca(CO_3_)		15.7		5.4
Biotite	KFeMg_2_(AlSi_3_O_10_)(OH)_2_	0.8	3.5		1.5
Cordierite	Mg_2_(Al_4_Si_5_O_18_)	0.9	1.5		
Dolomite	CaMg(CO_3_)_2_		5.7		

**Table 2:** Mix proportion of aeolian sand concrete (by mass).

Group ID	Cement	Aeolian Sand	Superplasticizer	Water
Tak	1	1.2	0.02	0.22
Gur	1	1.2	0.02	0.25
Kum	1	1.2	0.02	0.22
Bei	1	1.2	0.02	0.22
Bei-AA	1	1.2	0.02	0.22
Tak-CNTs	1	1.2	0.02	0.22
Gur-CNTs	1	1.2	0.02	0.25
Kum-CNTs	1	1.2	0.02	0.22
Bei-CNTs	1	1.2	0.02	0.22

**Table 3:** Summary of Experimental Methods, Specimen Preparation, and Equipment.

Test Item	Objective	Specimen Preparation and Pretreatment	Key Equipment and Main Parameters
Mechanical Properties	Determine compressive/flexural strength and energy dissipation	Compressive: 70.7 mm cubes; Flexural: 40 × 40 × 160 mm prisms. Three samples per group	Pressure testing machine (Zhejiang Yiyu Instrument Equipment Co., Ltd, Shaoxing, China); Loading rate: 0.5 MPa/s (compressive), 0.05 MPa/s (flexural)
X-ray Diffraction (XRD)	Analyze hydration products, chemical composition of sand, and ITZ crystal structure	Powdered samples.	Ultima IV (Rigaku, Tokyo, Japan); 40 kV, 40 mA, Cu target, 0.154 nm wavelength, scanning step 0.02°
Nanoindentation	Analyze Interfacial ITZ width and micro-hardness	3 mm fragments, hydration terminated in anhydrous ethanol (24 h), embedded in epoxy resin, ground and polished	Nanoindentation tester (KLA Corporation, Milpitas, CA, USA); Loading rate 0.25 mN/s, maximum load 2 mN, 5 × 10 spot array, 5 μm spacing
FTIR Spectroscopy	Detect chemical bonds between ssDNA and aeolian sand surface	Potassium bromide (KBr) tableting method	Nicolet iS5 (Thermo Scientific, Waltham, MA, USA)
Thermogravimetric Analysis (TGA)	Identify hydration product types and calculate hydration degree	Hydration terminated in anhydrous ethanol (7 d), ground to 200 mesh	Q600 SDT (TA Instruments, New Castle, DE, USA); RT to 1000 °C, 10 °C/min heating rate under nitrogen atmosphere
SEM/EDS	Observe ITZ porosity, microstructure, and CNT distribution	Fragmented specimens; EDS for elemental distribution analysis	JSM-7800F (JEOL, Tokyo, Japan); Operating voltage 20 kV
TOF-SIMS	Analyze new substances generated by ssDNA-sand reaction	Direct scanning of modified aeolian sand surfaces.	PHI Nano TOF II (ULVAC-PHI, Inc., Kanagawa, Japan); Bi^2+^ ion beam (30 keV), ion current 2 nA, raster size 200 × 200 µm
Transmission Electron Microscopy (TEM)	Analyze C-S-H crystal structure and stacking	ssDNA/CNT solution mixed with cement paste, ultrasonicated (20 min), deposited on 200-mesh copper grid	JEM-F200 (JEOL, Tokyo, Japan)
Mercury Intrusion Porosimetry (MIP)	Characterize nanoscales to microscale pore structure	1 cm^3^ fragments, hydration terminated in anhydrous ethanol (48 h), dried to constant weight	Auto Pore V9500 (Malvern Panalytical Ltd., Malvern, UK); Maximum mercury pressure 228 MPa

**Table 4:** ssDNA fingerprint peaks and corresponding functional groups in nucleotides.

Peak Number	Wave Number (cm^−1^)	Assignment	Nucleotide Component
1	835	δ(C-H)	Sugar
2	895	Deoxyribose	Sugar
3	921	ν(C-O-C)	Sugar
4	997	ν(C-O)	Sugar-phosphate
5	1030	ν(C-C) + ν(CH_2_OH) + ν(C-O) + δ(C-O)	Sugar-phosphate
6	1047	ν(C-C) + ν(C-O) + δ(C-O)	Sugar
7	1077	ν(PO_2_^−^)	Base-sugar
8	1106	ν(P=O) + ν(P-O-C)	Sugar-phosphate
9	1206	ν(P=O)	phosphate
10	1255	ν(P=O)	phosphate
11	1295	ν(C-O)	phosphate
12	1403	δ(C-H) + δ(N-H) + ν(C-N)	Base
13	1454	δ(C-H)	Base
14	1617	ν(C=O)	Base
15	1659	ν(C=O)	Base
16	1697	ν(C=O)	Base

Note: ν: tensile vibration; δ: bending vibration.

**Table 5:** Cost and carbon emission analysis of the prepared concrete vs. conventional C80 concrete.

This Study				C80			
Component	Usage Rate (kg/m^3^)	Cost (CNY/kg)	Carbon (kg CO_2_e/kg)	Component	Usage Rate (kg/m^3^)	Cost (CNY/kg)	Carbon (kg CO_2_e/kg)
Cement	1302.08	520.83	957.03	Cement	360.00	144.00	264.60
Aeolian sand	1562.50	0.00	0.00	Mineral powder	110.00	22.00	6.26
Water	299.48	0.90	0.05	Medium sand	700.00	84.00	1.76
Superplasticizer	26.04	234.38	70.31	Crushed stone	970.00	97.00	2.11
KOH	117.19	35.16	154.69	Water	138.00	0.41	0.02
CNTs	0.003	1.56	0.01	Fly ash	60.00	7.20	1.80
ssDNA	0.003	31.25	0.01	Superplasticizer	8.50	76.50	22.95
				Sucrose ester	3.00	60.00	0.00
				Polyacrylamide	1.50	9.00	0.00
				Benzoic acid	0.54	2.70	0.00
Total		824	1182			502	300

**Table 6:** Comparison of the advantages of this study over traditional nanomaterial modification.

This Study				GO [14]			
Component	Usage Rate (kg/m^3^)	Cost (CNY/kg)	Carbon (kg CO_2_e/kg)	Component	Usage Rate (kg/m^3^)	Cost (CNY/kg)	Carbon (kg CO_2_e/kg)
Cement	1302.08	520.83	957.03	Cement	1097	438.80	806.29
Aeolian sand	1562.50	0.00	0.00	GO	5.48	135,173	18.26
Water	299.48	0.90	0.05	Silica sand	548	4693	0
Superplasticizer	26.04	234.38	70.31	Hydrochloric acid	217	20,000	26.04
KOH	117.19	35.16	154.69	Water	439	1.32	0.07
CNTs	0.003	1.56	0.01	3-aminopropyltriethoxy silane	3.89	389	12.96
ssDNA	0.003	31.25	0.01	Alcohol	324	4402	648
Total		824	1182			165,097.12	1511.62

## Data Availability

The original contributions presented in this study are included in the article. Further inquiries can be directed to the corresponding author.
